# Variability of key-performance-indicators in commercial gilthead seabream hatcheries

**DOI:** 10.1038/s41598-022-23008-z

**Published:** 2022-10-25

**Authors:** Chara Kourkouta, Andreas Tsipourlianos, Deborah M. Power, Katerina A. Moutou, George Koumoundouros

**Affiliations:** 1 Biology Department, University of Crete, Vasilika Vouton, 70013 Heraklion, Crete Greece; 2 Department of Biochemistry and Biotechnology, University of Thessaly, 41500 Larissa, Greece; 3 Centre of Marine Sciences (CCMAR), University of Algarve, Faro, Portugal

**Keywords:** Ichthyology, Animal breeding

## Abstract

Skeletal abnormalities are one of the most important key-performance-indicators (KPIs) in finfish hatcheries. Coping with the problem of skeletal abnormalities relies on the understanding of the link between the variability in the rearing conditions, and the variability in abnormalities incidence. Here, 74 seabream larval populations, from four commercial hatcheries, were examined for the presence of abnormalities and monitored with respect to the applied conditions. The inward folding of gill-cover and pugheadedness were the most frequent abnormalities present, with a mean (± SD) frequency of 11.3 ± 17.9 and 6.0 ± 7.2%, respectively. Other abnormalities were observed at very low mean rates (≤ 1%). A new abnormality type, ray-resorption syndrome, was also found. The recorded rate of normally inflated swimbladder was 92.3 ± 7.4% and mean survival rate was 25.9 ± 21.0%. Classification tree analysis indicated six rearing variables as potentially important predictors for pugheadedness, six variables for caudal-fin abnormalities and 10 variables for survival rate. Complementary genetic analysis, revealed differentiating genetic diversity and significant genetic distances among participating hatcheries, suggestive of the role of company-specific management of genetic resources in KPIs’ variability. The results are discussed with respect to their potential use in the control of skeletal abnormalities by commercial hatcheries, as well as for benchmarking among different hatcheries.

## Introduction

Skeletal abnormalities are an important issue for the quality of reared fishes, with either an emerging^1–6^ or well-established production^7–11^. In most aquaculture species, skeletal abnormalities develop during mainly the embryonic and larval stages, i.e. the period of skeleton development^12^, and represent an important key-performance-indicator (KPI) for hatcheries. The extensive research efforts of the last 25 years have remarkably enhanced knowledge on the causative factors of abnormalities in reared fish. Currently a variety of nutritional components during the larval period (e.g. fatty acids^13^, vitamins^14,15^, weaning method^16^), abiotic parameters (e.g. water temperature^17^, tank color^18^, hypoxia and hypercapnia^19^), the intensity of the rearing methodology^20,21^ and genetic background^9,22,23^ have been shown to play a significant role in the development of skeletal abnormalities in reared fish (see Boglione et al.^24^ for a thorough review). Different abnormality types develop during different ontogenetic windows, and presumably under the action of different causative factors^12^.

Skeletal abnormalities present a wide range of phenotypic expression, from abnormalities of a few internal skeletal elements to more severe abnormalities with light or pronounced effects on the external phenotype of fish. Interestingly, some abnormality types may recover to a greater^25^ or lesser^26^ extent during the on-growing period, through a process that may involve remodeling of the abnormal bones^25^. Skeletal abnormalities may significantly affect the external morphology^27,28^, and thus lower the commercial value of fish that are marketed whole. Furthermore, some abnormality types can induce severe mortality^29^, decrease the growth rate or increase the susceptibility of fish to diseases (reviewed by Boglione et al.^24^). To minimize these negative effects, it is common practice in commercial hatcheries to remove abnormal fish from the reared stocks at the end of the hatchery phase, well before they reach the final consumer.

Despite substantial gains in knowledge about the causative factors of skeletal abnormalities, their presence is a persistent problem in commercially reared fish stocks. The failure to resolve this issue is multifactorial and may be attributed to difficulties in knowledge transfer from the research to applied level, poor knowledge about the action of some critical factors, inappropriate management of genetic resources, accidental deviations from standard hatchery operating procedures, as well as inadequate control of variations in critical rearing parameters or raw materials for feed. As a result, the incidence and typology of skeletal abnormalities may be highly variable among different hatcheries, as well as among fish batches from the same hatchery^12,24,29^. In any given hatchery, intra-batch variability lowers the consistency of fish quality, hatchery productivity and animal welfare. To our knowledge, the variability of skeletal abnormalities in commercial hatcheries has been examined in only a limited number of studies. Cobcroft and Battaglene^30^ examined the rates and abnormality type in samples of four species taken from Australian commercial hatcheries. Prestinicola et al.^31^ examined the variability of skeletal abnormalities in eight gilthead seabream larval populations which were reared in one commercial (six populations) and one research (two populations) hatchery. Recently, Cavrois-Rogacki et al.^27^ examined the variability of skeletal abnormalities in 12 populations of ballan wrasse (*Labrus bergylta*) that were reared in two commercial hatcheries.

Coping with the problem of skeletal abnormalities at the level of commercial hatcheries, is to a great extent dependent on understanding the link between variability in rearing processes and the variability in fish quality. Therefore, it is essential that appropriate monitoring of rearing parameters and fish quality control are performed simultaneously, at the level of single populations (i.e. before different populations are mixed). In the present study we aimed to (a) identify and characterize the currently most important abnormalities in four gilthead seabream commercial hatcheries, (b) study the variability of biological KPIs in commercial hatcheries in order to estimate reference values for benchmarking and c) identify potentially critical parameters for their contribution to the observed KPIs variability. The current genetic structure within the studied hatcheries is also considered since it is an important factor that is expected to introduce variability in all biological KPIs. The advent of breeding programs combined with the exchange of genetic material between farmers and rearing practices have most likely changed the genetic structure in the hatcheries compared to earlier studies^32,33^. Microsatellite markers (SSR) were used to map the current genetic structure of larval populations since they are neutral and sensitive markers for detecting genetic variation within and between differentiated populations^34^. In total, four commercial hatcheries, located in three EU Mediterranean countries, were included in the analysis.

## Results

### Rearing methodology

Eggs were taken from breeders of 4.2 ± 0.9 kg weight, held in indoor tanks at a sex ratio (female to male fish) of 2.8 ± 2.8. Breeders were fed 3–5 days per week on dry pellets and occasionally on frozen fish. All participating hatcheries reported that broodstock was part of an on-going selective breeding program (Table 1). Eggs were concentrated in 50–250 L surface-water collectors connected on the outlet of the broodstock tanks. Eggs were removed from the egg collectors on average at noon (13:40 ± 6:20) and usually (in 75% of the cases) subjected to disinfection with certified chemical products. The embryonic stage was performed either in egg incubators (in 57% of the cases), or directly in the larval rearing tanks (in 43% of the cases). The mean reported rate of egg fertilization was 84.8 ± 4.9%. Table 1 summarizes all the rearing conditions which were monitored during the broodstock management and embryonic period.

**Table 1:** Range of the main rearing parameters during the broodstock management and embryonic period.

Parameter	mean ± SD	min	max	n
**Broodstock**				
Mean age of breeders (yr)	4.2 ± 0.9	3	6	68
Mean size of breeders (Kg)	1.9 ± 0.4	1.5	3.2	68
Sex-ratio (F/M)	2.8 ± 2.8	0.8	11.0	64
Water temperature (°C, egg collector)	18.6 ± 0.4	17.4	19.5	68
O_2_ concentration (mg/L, egg collector)	9.0 ± 0.6	7.5	9.6	29
Salinity (egg collector)	35.0 ± 1.8	30.2	37.0	68
pH (egg collector)	7.7 ± 0.3	6.7	7.9	48
Volume of the egg collector (L)	183 ± 84	50	250	68
Diet	Mixed^a^ or pellet-only			
Feeding rate	Ad libitum			
Feeding frequency	3–5 days per week			
Active selective breeding program?	Yes (68/68)			
**Embryonic period**				
Time at egg collection	13:40 ±	8:00	23:00	64
Fertilization rate (%)	84.8 ± 4.9	75.0	95.0	49
Egg disinfection	Yes (75%, 51/68)			
Use of egg incubator	Yes (57%, 42/74)			
Volume of the egg incubator (L)	959 ± 876	140	1950	42
Stocking density in the incubator (eggs/L)	3641 ± 2507	600	8164	42

n, number of reported values (cases).

^a^Mixed diet, with use of frozen fish (e.g. sardines, squids) and commercial pellets.

**Figure 1:**
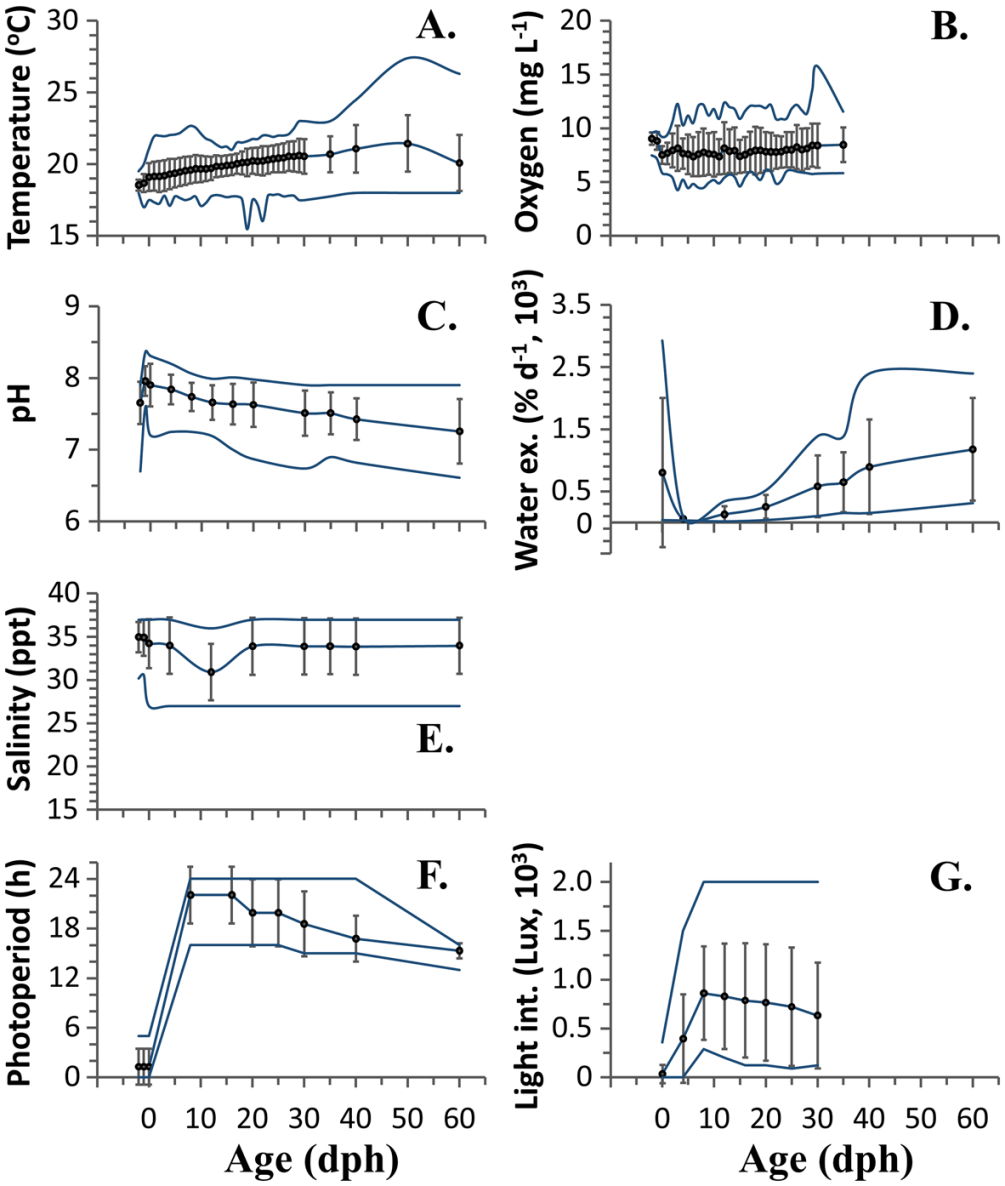
Mean (± SD), min and max values of the basic abiotic conditions applied during the embryonic period and larval rearing of gilthead seabream in 4 European hatcheries. (**A**) Water temperature (°C). (**B**) Oxygen concentration (mg/L). (**C**) pH. **D.** Daily water exchange rate (% of tank volume). (**E**) Salinity (ppt). (**F**) Photoperiod (duration of light, hours). (**G**) Light intensity on the water surface (Lux). dph, days post hatching.

Larval rearing was performed in cylindrical tanks (10–25 m^3^ volume, 0.5–2.2 m depth) with black, green or yellow walls (Table 2). Larval rearing was performed in flowthrough systems, with water pumped from boreholes or the open sea, and treated with UV and/or ozone. Mean water temperature gradually increased from 18.5 ± 0.4 °C at spawning, to 19.1 ± 1.0 °C at hatching and 21.4 ± 2.0 °C at 50 dph (Fig. 1A). Mean oxygen concentration was almost stable during the larval period (Fig. 1B), whereas mean pH decreased (Fig. 1C) and water exchange rate increased (Fig. 1D). Mean water salinity was generally stable during the studied period, with a short drop at ca 12 dph (Fig. 1E). Egg incubation and yolk-sac larval stage were performed in the dark, whereas at feeding onset a 16:8 to 24:0 h L:D photoperiod was applied (Fig. 1F,G).

**Table 2:** Range of the main rearing parameters during the larval-rearing period.

Parameter	mean ± SD	min	max	n
**Larval rearing**				
Tank volume (m^3^)	11.8 ± 4.1	10	25	74
Tank depth (m)	1.3 ± 0.4	0.5	2.2	55
Tank shape	Cylindrical (74/74)			
Tank colour^a^	B (53/74), G (18/74), Y(3/74)			
Number of tanks in the same room	14 ± 8	5	36	73
Tank disinfection prior stocking	Yes (74/74)			
Duration of dry period prior stocking (d)	6.6 ± 7.5	1	31	68
Water source	Borehole (55/74), open-sea (19/74)			
UV water-treatment	Yes (89%, 66/74)			
Water Ozonation	No (85%, 63/74)			
Use or RAS for the larval rearing	NO (74/74)			
Use or RAS for the weaning	Yes (77%, 57/74)			
Initial stocking density (fish/L)	152 ± 61	70	330	74
Use of eggs of different ages	No (95%, 70/74)			
Onset of siphon cleaning (dph)	9.3 ± 3.5	0	13	74
Age at fish removal from the larval-rearing tank (dph)	44 ± 13	26	70	73
Total rotifers (10^6^)	6968 ± 3746	2366	21,336	74
Total Artemia instar I nauplii (10^6^)	4572 ± 1664	1625	9909	72
Total Artemia instar II nauplii (10^6^ )	283 ± 211	0	752	74
Rotifer adjustment levels (ind/mL)	4–12			
Artemia instar I adj. levels (ind/mL)	0–3			
Artemia instar II adj. levels (ind/mL)	0.25–5			
Daily number of algae inputs	1 to continuous			
Daily number of rotifers provisions	2 to continuous			
Daily number of Artemia provisions	5 to continuous			
Algae used^b^	Iso, Nan, Chl, Nps, other			
Use of more than one algal species	No (78%, 58/74			
Number of enrichment prod. used (rotifers)	One (39/74) to two (35/74)			
Number of enrichment prod. used (Artemia)	1 (74/74)			

**Figure 2:**
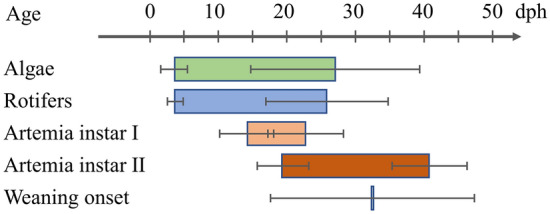
Mean duration of live feed provision (± SD, standard deviation) during the larval rearing of gilthead seabream in four European hatcheries. dph, days post hatching.

For the rest abiotic and nutritional parameters, see also Figs. 1, 2, S1.

^a^*B* black, *G* green, *Y* yellow.

^b^Iso *Isochrysis galbana*; Nan *Nannochloris atomus*; Chl *Chlorella minutissima*; Nps *Nannochloropsis* sp.

Larvae were reared at an initial stocking density of 152 ± 61 fish L^−1^ (Table 2) in the presence of background phytoplankton (3 ± 2 to 27 ± 12 dph, Fig. 2). According to the average feeding protocol which was applied, larvae were initially fed on enriched rotifers (4 ± 1 to 26 ± 9 dph), whereas in the next period Artemia instar I (14 ± 4 to 23 ± 5 dph) and enriched Artemia instar II nauplii (19 ± 4 to 41 ± 12 dph) were also provided. Weaning started on average at 32 ± 15 dph (Fig. 2). At 44 ± 13 dph fish were removed from the larval rearing tanks and transferred to weaning or pre-growing facilities. Further details on the larval rearing parameters are given in Table 2. The mean quantities of live and dry feeds provided to each larval population are given in Fig. S1.

### Key-performance-indicators

**Figure 3:**
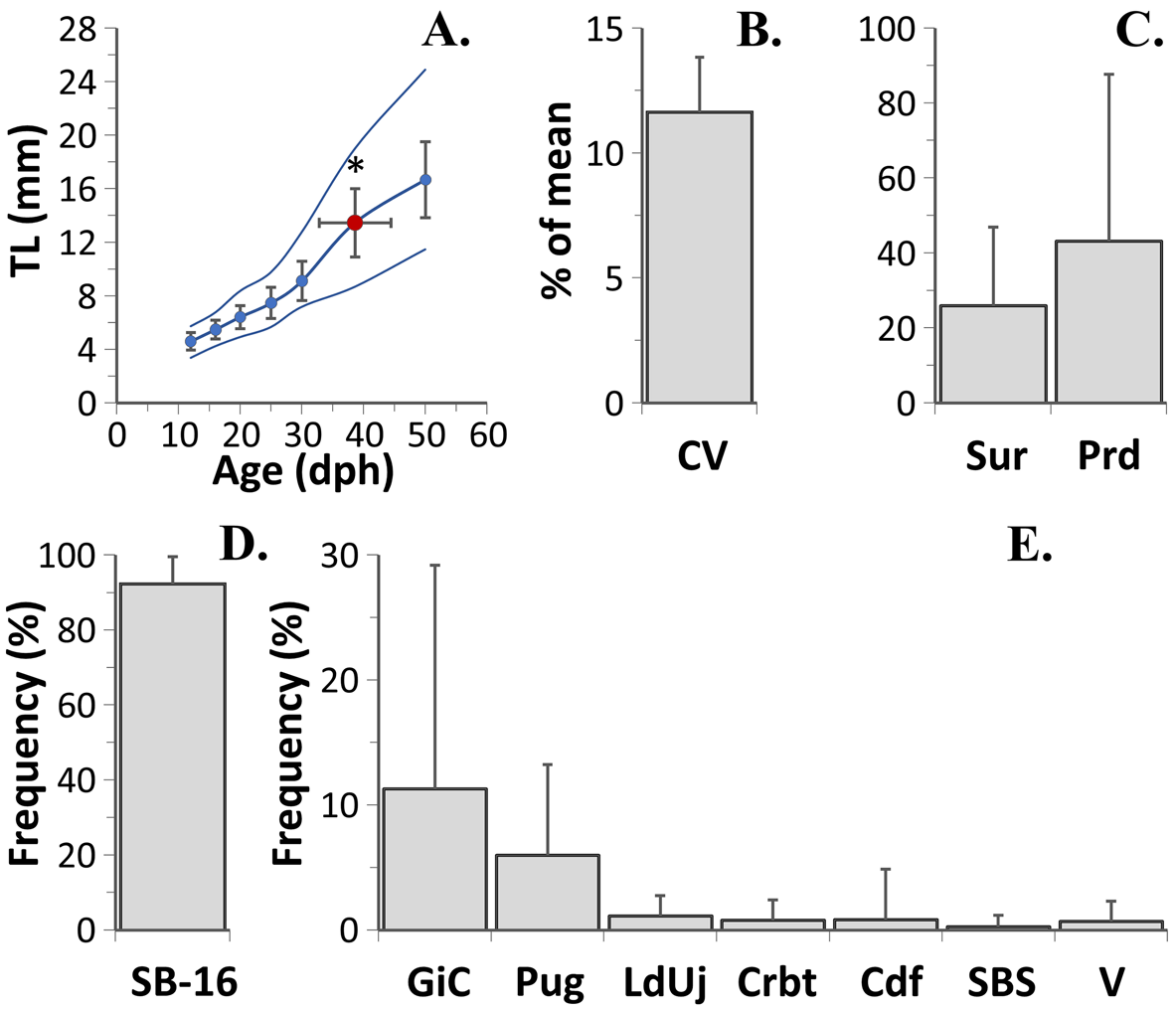
Mean values of the recorded Key-Performance-Indicators in the 74 monitored populations. (**A**) Average fish total length (TL) during the larval rearing and weaning period. Asterisk (*) indicates the mean TL and age of the fish samples which were examined for skeletal abnormalities (quality control). (**B**) Coefficient of variation of fish TL at quality control. (**C**) Average survival rate (Sur, in %) at 26–70 dph. Prd indicates the mean number of fish survived per volume unit (L) of the larval-rearing tank (fish/L). (**D**) Mean frequency of normally inflated swimbladder at 16 dph. (**E**) Mean frequency of skeletal abnormalities. Cdf, caudal-fin abnormalities. Crbt, crossbite. GiC, gill-cover abnormalities. LdUj, lateral displacement of the upper jaw (fused maxillaries and pre-maxillaries). Pug, pugheadedness. SBS, saddleback syndrome. V, vertebral abnormalities. Error bars equal 1 standard deviation. n = 74 samples (of ca 50 specimens each).

Fish size increased from 4.6 ± 0.7 mm TL at 12 dph to 16.7 ± 2.8 mm TL at 50 dph (Fig. 3A). At quality control (39 ± 6 dph) mean TL was 13.5 ± 2.6 mm (CV = 11.6 ± 2.2%, Fig. 3B) and SGR was 0.038 ± 0.003 d^−1^. At the end of the larval rearing phase, the reported mean survival rate was 25.9 ± 21%, with 43.2 ± 44.5 fish produced per L of larval-rearing tank volume (Fig. 3C). The mean rate of normally inflated swimbladder was 92.3 ± 7.4% (Fig. 3D).

**Figure 4:**
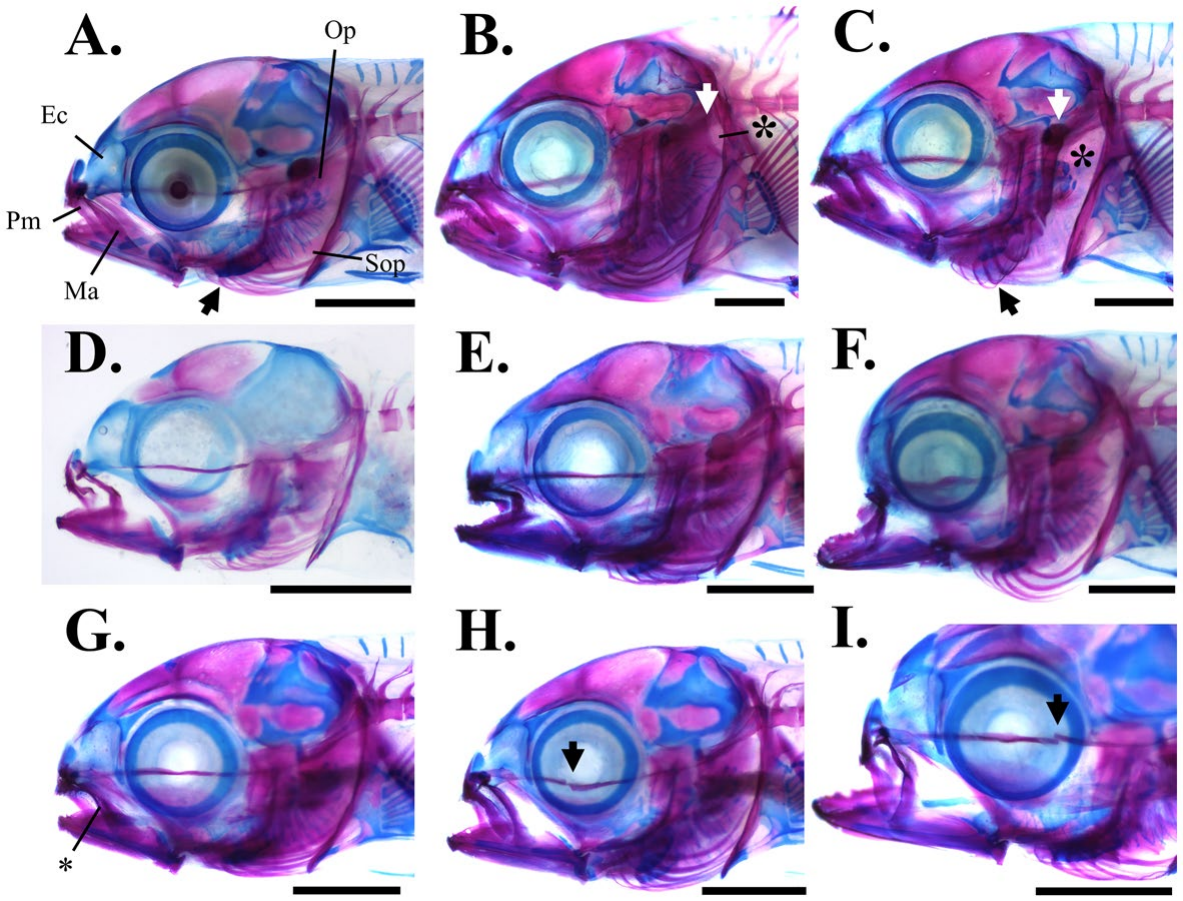
Variability of cranial abnormalities in the examined samples. (**A**) Normal. (**B–C**) gill-cover abnormality, light (**B**) and severe (**C**), with inward folding of the operculum (white arrow), sub-operculum and branchiostegal rays (**C**, black arrow). Asterisks indicate the gill-chamber. (**D-E**) light (**D**) and severe (**E**) pugheadedness, associated with twisted maxillaries and pre-maxillaries. (**F**) Severe pugheadedness, with lack of pre-maxillaries, malformed maxillaries, ethmoid cartilage and lower jaw. (**G**) Fusion (*) between the maxillary and pre-maxillary bones. (**H–I**) Pugheadedness, associated with the fracture of the parasphenoid-vomer bar (arrows). Ec, ethmoid cartilage. Ma, maxillary. Op, operculum. Pm, pre-maxillary. Sop, sub-operculum. Scale bars equal to 1 mm.

A variety of skeletal abnormalities were recorded in the examined samples, with a variety of severity degrees. The inward folding of gill cover (Fig. 4B–C; Fig. 4A shows the normal anatomy) was the most frequent abnormality in the examined samples (11.3 ± 17.9%, Fig. 3E). It was followed by the compression of the ethmoid area and upper jaws (pugheadedness, 6.0 ± 7.2%, Fig. 3E), which was mainly associated with abnormalities of the maxillaries and pre-maxillaries (Fig. 4D–F), and to a lesser extent with fracture of the parasphenoid-vomer bar (Fig. 4H–I). Other abnormalities were observed with a small mean incidence (Fig. 3E) and affected the upper jaws (fused maxillaries and pre-maxillaries, 1.1 ± 1.6%, Fig. 4G), the lower jaw (crossbite, 0.8 ± 1.6%), the caudal fin (e.g. shortening, stricture, duplication, 0.8 ± 4.1% Fig. S2) and dorsal fin (pterygiophore abnormalities, saddleback syndrome, 0.3 ± 0.9%, Figs. S3A, S3B), as well as the vertebral column (abnormalities of vertebral centra without associated axis deviations, kyphosis, lordosis, 0.7 ± 1.6%, Figs. S3C, S3D).

**Figure 5:**
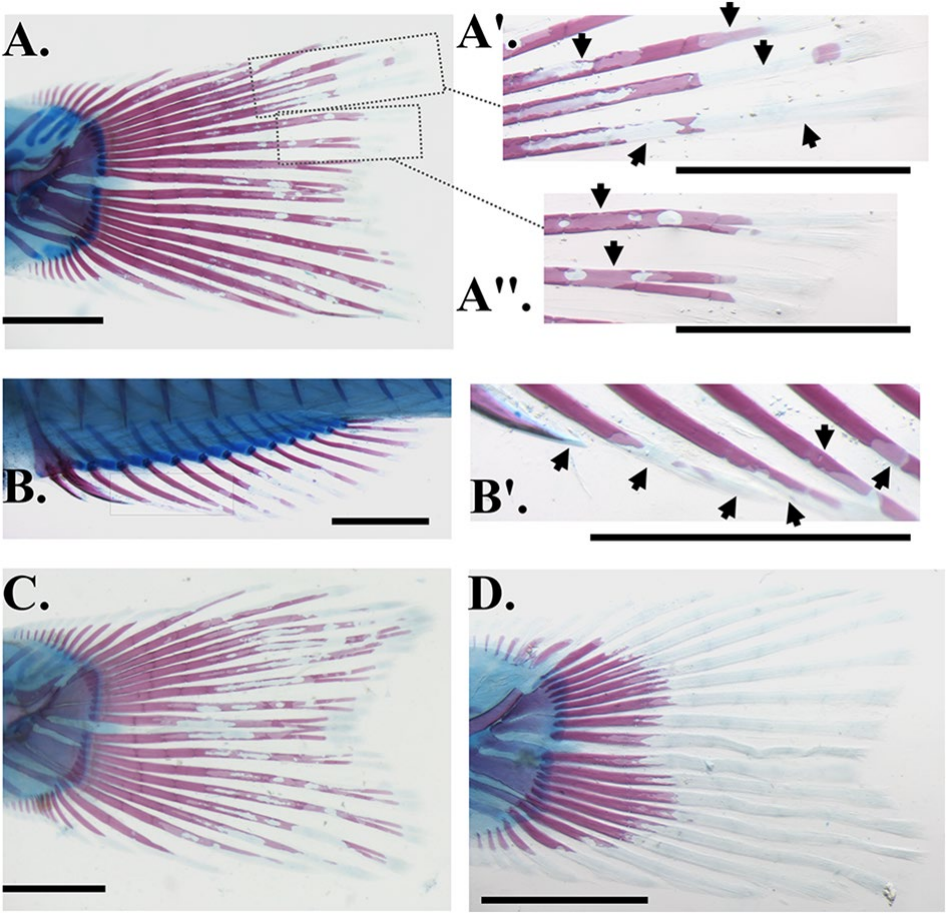
Representative examples of ray-resorption syndrome in seabream samples. (**A**) Caudal fin. (**B**) Anal fin. **A′–A′′** and **B′**. Insets of figure (**A**) and (**B**), showing in detail the non-mineralized areas, resembling typical resorption lacunae (arrows). (**C**, **D**). Examples of different phenotypes of ray-resorption syndrome on seabream caudal fin. Scale bars equal to 1 mm.

A new abnormality type, ray-resorption syndrome (RRS), was recorded in 7 out of 74 examined larval populations at a high incidence (ca > 90% in each sample). The RRS was macroscopically evident on the spines and lepidotrichia of all the fins, in the form of an irregular, mosaic ossification pattern (Fig. 5A–D). The detailed examination of the abnormal specimens revealed that the abnormal phenotype was linked to the presence of non-mineralized areas, resembling typical resorption lacunae, scattered along the lepidotrichia and spines. On the lepidotrichia, these non-mineralized areas could be present on one or both of the hemirays (Fig. 5A′,A′′ and B′). No obvious changes were observed on the non-mineralized matrix and the connective tissue of the lepidotrichia. RSS was not associated with abnormalities of the ray supporting skeletal elements (e.g. hypurals, pterygiophores etc.).

### Potentially critical factors for KPIs variability

For all the KPIs and hatcheries examined, CTa indicated a total of 25 variables as important predictors for the independent variables (KPIs). Of these 25 variables, 10 were related with abiotic and 14 with nutritional parameters. Stocking density at the beginning of larval rearing phase was the other important predictor indicated (Table 3). Abiotic predictors mainly concerned fluctuations of water temperature or oxygen concentration, expressed either as standard deviations of the daily mean or as the sum of daily ranges (max–min) during certain periods (Table 3). Nutritional predictors mainly concerned the quantity of rotifers, Artemia nauplii or dry feed provided during certain periods of the larval rearing phase (Table 3).

**Table 3:** Critical factors for certain KPIs in the different hatcheries, as indicated by classification tree models.

KPI	Potentially critical factor	Rel	nH	nL	Cor. Clas. (%)	Sign
Pug	Total Rotifers (3–35 dph)	N	10	9	89.5	**
	Artemia instar II (15–36 dph)	N	10	9	94.7	**
	Artemia instar II (26–36 dph)	N	10	9	94.7	**
	Artemia instar II (26–36 dph)	N	9	8	100.0	**
	Artemia instar II (30 dph)	N	10	9	89.5	**
	SD of temperature (10–14 dph)	N	9	8	100.0	***
Cdf	O_2_ cum. differences (daily, 11–20 dph)	P	9	8	100.0	***
	O_2_ SD (10–14 dph)	P	9	8	100.0	***
	Temperature cum. differences (daily, 0–10 dph)	N	9	8	100.0	***
	Artemia instar II (15–36 dph)	N	9	8	100.0	**
	Age @ Weaning onset (dph)	N	9	8	100.0	***
	Rotifer adjustment-levels	N	9	8	100.0	***
SBS	Total Dry Feed (21–25 dph)	N	14	5	100.0	***
	Artemia instar II (15–36 dph)	P	8	9	82.4	–
V	SD of temperature (25–29 dph)	P	10	8	88.9	**
Sur	Rotifers (3 dph)	P	9	10	100.0	**
	Artemia instar II (15–36 dph)	P	9	8	94.1	***
	Age @ Weaning onset (dph)	P	9	8	94.1	***
	Initial Stocking Density	N	9	8	100.0	***
	SD of temperature (0–4 dph)	N	9	8	88.2	**
	SD of temperature (5–9 dph)	N	9	9	94.4	**
	SD of temperature (15–19 dph)	N	9	8	88.2	*
	Artemia instar I (product name)		9	8	88.2	-
	Air-diffusers (number)	N	12	8	100.0	***
	Spawning temperature	P	12	8	95.0	***

“Rel” column indicates the type of relationship between KPIs and critical factors (P, positive; N, negative), “nH” and “nL” columns the count of cases with high and low quality respectively, “Cor. Clas.” the % of correct classifications, and “Sign.” the significance of the difference in the critical factors between the nH and nL groups (*p < 0.05; **p < 0.01; ***p < 0.001). Analyses were performed separately for the different hatcheries. Cdf, caudal-fin abnormalities. Pug, pugheadness. SBS, dorsal-fin abnormalities. Sur, survival rate at 26–70 dph. V, vertebral abnormalities. KPI to critical factor association is purely statistically based on the specific data provided. They can serve only in building hypotheses to be tested by each hatchery.

Following CTa, the incidence of pugheadedness, the second most frequent abnormality type, was negatively related with the total quantity of rotifers (3–35 dph), or Artemia instar II nauplii provided during certain rearing periods (26–36 dph in the case of two hatcheries, 15–36 dph, 30 dph in the case of single hatcheries). No predictor was indicated for gill-cover abnormalities, the most frequent abnormality type, whereas six predictors were indicated for caudal-fin, two for dorsal-fin and one for vertebral abnormalities (Table 3). Ten predictors were indicated as important for the fish survival rate at 26–70 dph. In three of these cases, survival rate was negatively related with the SD of water temperature during certain rearing periods (0–4, 5–9 or 15–19 dph), as well as with the fish stocking density at the beginning of larval rearing. The survival rate was positively related with the quantity of rotifers and Artemia instar II provided at 3 dph and 15–36 dph, as well as with fish age at weaning onset (Table 3).

### Hatchery genetic diversity

**Figure 6:**
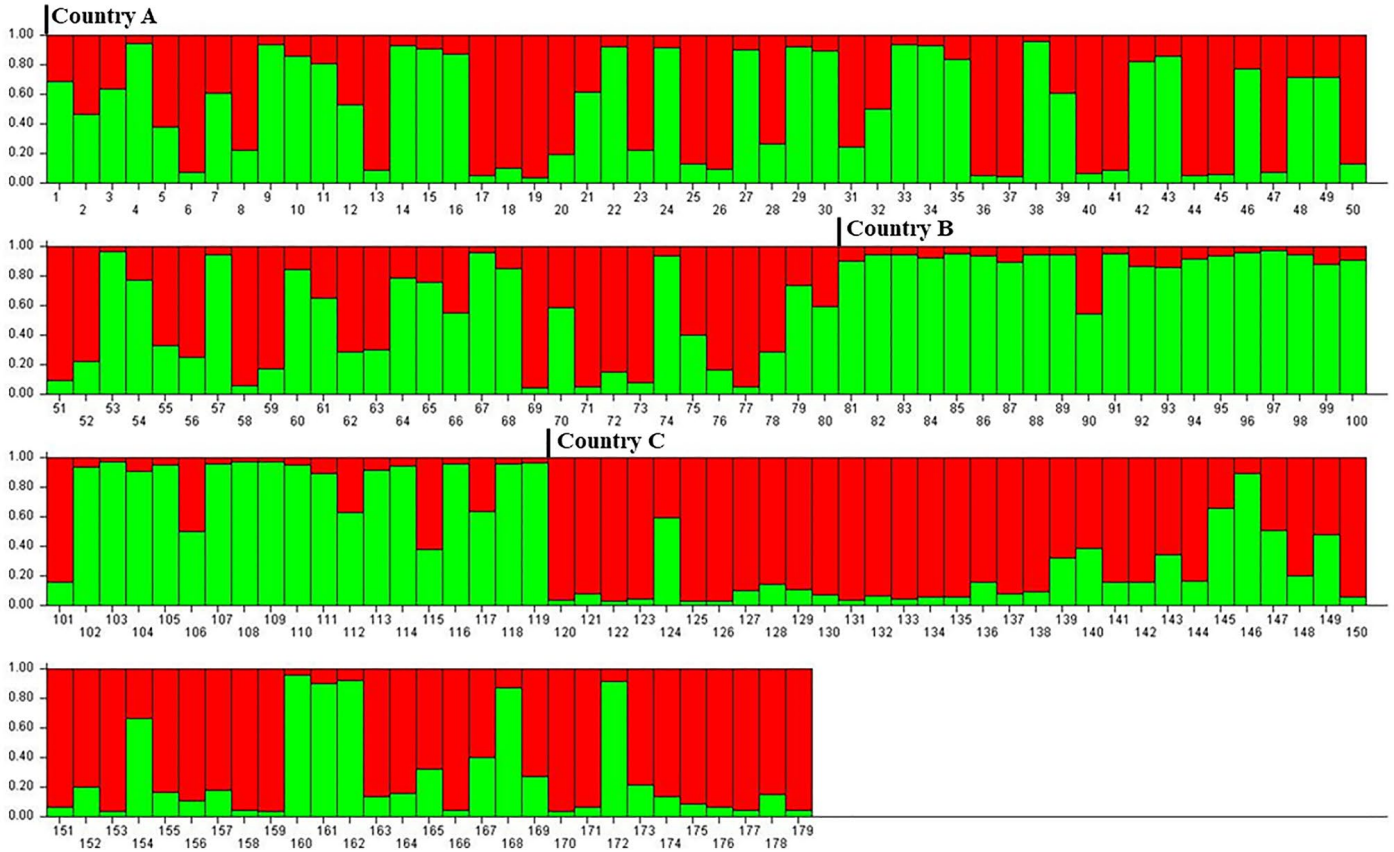
Genetic variability and population structure of the hatchery populations under study. For an overall estimated K = 2 (red, green), the admixture pattern of each individual larvae sample analysed is provided to indicate the contribution of each of the two genetic origins (K). A clear differentiation of admixture patterns is evident by country, with a domination of one origin (green) in country B and the domination of the other origin (red) in country C.

All SSRs exhibited a high degree of polymorphism with 10–15 alleles recorded per SSR (Table 4A). However, the number of alleles recorded per SSR varied between hatcheries (Table 4A). The observed heterozygosity values were low considering the number of alleles identified in each SSR (Table 4A). F_ST_ values and Nei genetic distances indicated significant genetic differentiation between the four hatcheries (Table 4B). In addition, best K values, calculated by plotting the second order of change of L(K) (Δ(Κ)), ranged between 2 and 4 among hatcheries, indicative of the genetic variation hosted in each hatchery. In all hatcheries considered, K = 2. Plotting the q-values for each of the individual gilthead seabream samples analyzed, indicated that the admixture patterns differentiated between the countries the four hatcheries were located in (Fig. 6).

**Table 4:** (A) Allele number, observed heterozygosity (Ho) and expected heterozygicity (He) for each of the four loci in the samples provided by the four participating hatcheries; (B) Pairwise *F*ST (above diagonal) and pairwise *Nei’s* genetic distances (below diagonal), between the four hatcheries based on the analysis of four microsatellite loci.

(A)				
	SAIMB26	SAUK14INRA	FD-78-H	Sal12
Range of allele number	6–12	7–10	5–15	6–12
Mean number of alleles	*10.0*	*8.50*	*9.25*	*8.00*
Range of H_o_	0.55–0.80	0.73–0.80	0.60–0.72	0.62–0.90
*Mean H*_*o*_	*0.66*	*0.74*	*0.71*	*0.70*
Range of H_e_	0.82–0.84	0.76–0.86	0.74–0.88	0.76–0.85
*Mean H*_*e*_	*0.82*	*0.81*	*0.84*	*0.79*

(B)				
	Hatchery A	Hatchery B	Hatchery C	Hatchery D
Hatchery A		0.051***	0.063***	0.026***
Hatchery B	0.223		0.039***	0.067***
Hatchery C	0.366	0.335		0.074***
Hatchery D	0.114	0.273	0.367	

Asterisks indicate statistical significance at *p* < 0.001. Mean values are in italics.

## Discussion

In the present study, 74 seabream larval populations, from four different commercial hatcheries, were examined for the presence of skeletal abnormalities and monitored with respect to the applied rearing conditions. Emphasis was given to analysis of the embryonic and larval stages (up to 39 ± 6 dph) because most skeletal abnormalities develop during these stages^12^. Analysis did not include haemal lordosis, the only known abnormality that may develop during the late metamorphosis and early juvenile period in a variety of fish species^35–37^ including gilthead seabream^38,39^. No comparisons of fish quality between the different hatcheries were performed in the present study, due to the large intra-hatchery variability in the incidence and typology of skeletal abnormalities^24^ and the relatively small number of larval populations analyzed from each hatchery (17–20). The results of the present study showed that the inward-folded gill cover and pugheadedness were the most frequent skeletal abnormalities in the examined samples, whereas other cranial, fin and vertebral abnormalities presented very low mean rates (≤ 1%, Fig. 3).

Gill-cover abnormalities have been shown to develop in many different fish species under experimental or commercial hatchery conditions^24,27^. The existing literature shows that their development may be induced by suboptimal levels of vitamin A (*S. aurata*^14^, *Dicentrarchus labrax*^40^), EFA, DHA and vitamin C (*Chanos chanos*^41,42^) in the larval diet, as well as by water temperature during the larval rearing phase (*S. aurata*^38^), or an early shift from live feed to compound diet (laboratory fish, *Danio rerio*^16^). Similarly, literature on the causative factors of pugheadedness in reared fish indicates the dietary levels of vitamin A (*S. aurata*^14^) and of n-3 PUFA (EPA, DHA) during the larval phase^40,43^ (*D. labrax*^44^) as critical. Studies on the causative factors of the other skeletal abnormalities observed, are mainly limited to those on the effects of vitamin A levels in the larval diet (*S. aurata*^14^) and water temperature during the embryonic and larval stages (*S. aurata*^17,38^, *Pagellus erythrinus*^45^) on the incidence of caudal-fin abnormalities.

In the present study, the intra-hatchery variability of skeletal abnormalities was examined simultaneously with the variability of the applied rearing methodology (150 variables). Classification tree analysis (CTa) indicated a total of fifteen rearing variables as potentially critical for the variability of abnormalities rate. The variability of pugheadedness and caudal-fin abnormalities was mainly linked with the variability of live feed provided during certain rearing, and to a lesser extent with temperature or oxygen fluctuations. Existing literature does not contradict the involvement of the most of these parameters in the development of pugheadedness and caudal-fin abnormalities, especially under the prism of ontogenetic shifts in fish nutritional preferences^46^. Our findings however on the decrease of the abnormality rate (both pugheadedness and caudal-fin) with the increase in temperature fluctuations during the early larval period (up to 14 dph, Table 3) cannot be supported by the existing literature. These results might possibly be explained by the decrease in fish survival rate with the increased temperature fluctuations (present study, Table 3) and the associated increase in food availability for the rest of the population.

In all hatcheries examined, CTa failed to indicate any predictor for the variability of gill-cover abnormalities, the most frequent abnormality type in the examined samples (present study). This result could be attributed to the action of factors other than those monitored in the present study, or might indicate the need for a larger number of examined populations from each hatchery. Our analysis (CTa) did not pool data from the different hatcheries because differences between hatcheries in not monitored parameters (e.g., operational or facilities related) could mask the outcome. On the other hand, this approach limited the case number and did not allow the validation of the CTa results by a dataset (testing) that was not included in initial analysis (model training^47^). At the practical hatchery level, on-site trials are required for the validation of the formed hypotheses (present study) and the following appropriate control of critical parameters.

In the last two decades, an increasing number of studies have highlighted the significance of fish genetic background for the development of skeletal abnormalities^48–50^. In gilthead seabream stocks, existing studies demonstrate that the genetic component of the variation in the development of abnormal gill-cover and pugheadedness varies from negligible to highly significant^23,51–53^. Of the rest abnormality types detected in the present study, generally at low incidence, namely lateral displacement of the upper jaw and caudal-fin abnormalities were recently shown to have a significant genetic component^9,23^. The diversity of fish farms and production strategies makes obtaining knowledge about the population structure a difficult task. This is because frequently the broodstock may be of unknown origin, there is an active exchange of eggs and juveniles across the Mediterranean, crossbreeding with wild individuals occurs and breeding programs are mostly company-specific. Recent studies provided evidence of significant genetic differentiation of wild stocks^54^ and wild vs farmed populations of gilthead seabream^34^. The results of the present study revealed a different degree of genetic variation hosted in the participating hatcheries, tracing back to two (2) populations of origin (K values). This finding, coupled with the low observed heterozygosity in relation to the number of alleles recorded for each SSR and the significant genetic distances recorded between hatcheries, are indicative of a reduction in genetic diversity in farmed gilthead seabream, most probably the result of genetic funneling achieved through directional selective breeding programs operated at a company level. In addition, the comparison of the admixture patterns of the individual samples was suggestive of a trend for differentiating admixture patterns according to the country in which the hatcheries were located. Based on the above observations we suggest that the genetic structure of the gilthead seabream in the hatcheries under study could be the result of a combination of two main factors: (a) an exchange of broodstock between breeders in previous times that formed the basis of the hatchery genetic variability, and (b) company-specific selective breeding programs that have been reducing the genetic diversity in a company-hatchery specific way and shaping the observed differentiation. We speculate that the company-specific management of genetic resources may have a role in the variability of KPIs.

Part of the solution for the problem of skeletal abnormalities in reared fish relies on our knowledge about their ontogeny and anatomy, and on the following effective hypothesis formation on the responsible causative factors^12^. Despite the relatively long list of studies in this field (reviewed by Boglione et al.^24^), new abnormality types may always appear in reared stocks^29^. In the present study, a new abnormality type (ray-resorption syndrome, RRS) was found to affect the ossification pattern of lepidotrichia and spines. The lack of an obvious link between RSS and abnormalities of the fin supporting elements, together with the normal shape and size of the affected rays, suggests that RSS developed after the formation of these skeletal elements. More studies, with samples at different ontogenetic stages, are required to address whether RSS may induce long-lasting changes in fish morphology, or whether the mineralization pattern of fin rays recovers in the next stages. In future, it will be interesting to assess if RRS is a typical bone resorption condition (removal of matrix and minerals), or if it is associated with the removal of minerals only.

Linking the results of fish quality control with precise and detailed records of the applied rearing conditions and genetic background, is fundamental in coping with the problem of skeletal abnormalities in the commercial hatcheries. Efforts should focus especially on the critical periods for the development of skeletal abnormalities and exploit all possible information on KPI variability. Data mining techniques can effectively be used to identify the potentially critical parameters for the control of abnormalities in each hatchery facility. The value of the results can be significantly increased by the inclusion of a larger number (if not all) of examined populations in the analysis. From this perspective, further support will be acquired by the automation of parameter recording, as well as by the incorporation of the required analytical tools in the management software used by the different hatcheries. The application of standard diagnostics at appropriate developmental periods, is a prerequisite for establishment of a benchmarking system between different production periods of the same hatchery, as well as, between hatcheries (with anonymized data, for policy making).

## Conclusions

The present study documented the variability of biological KPIs and rearing methodology in four commercial gilthead seabream hatcheries. Results demonstrated a large variability in the most of the examined KPIs and applied rearing conditions. Classification tree analysis was effective in identifying a list of potentially important predictors for particular types of skeletal abnormalities and survival rate. On-site trials for hypothesis testing and the monitoring/analysis of adequate number of larval populations are proposed as significant tools for controlling KPIs variability and improving the quality of fish in commercial hatcheries.

## Materials and methods

### Sampling and data collection

From each of the four participating hatcheries, 1–3 populations per month were randomly selected across a full production year, to be monitored for their rearing parameters and examined for the presence of skeletal abnormalities. A total of 74 (17–20 per hatchery) populations were analyzed. A list of larval-rearing parameters to be recorded was prepared and sent to the participating hatcheries. Requested data concerned fish nutrition, abiotic conditions, tank characteristics, and application of specific procedures (e.g., tank disinfection and cleaning methodology, egg disinfection), from broodstock maturation and egg collection, to egg incubation, larval rearing period, weaning and pre-growing phases (Tables 1, 2, S1, S2).

As the majority of skeletal abnormalities in gilthead seabream hatcheries develops during the embryonic and early larval period^12^, samples for quality control were taken before different larval populations were mixed together for the next rearing phase [26–70 days post-hatching, (dph); 9–19 mm mean total length, (TL)]. From each examined population, a random sample of 50–100 larvae was anaesthetized (ethylenglycol-monophenylether, 0.2–0.5 ml L^−1^) and fixed in phosphate buffered 5% formalin (pH = 7.2). Upon their arrival in the lab, all samples were anonymized and coded. Following the request of the commercial hatcheries, all data were handled exclusively by the University of Crete (laboratory of Marine Biology) and treated as strictly confidential and anonymous.

### Quality control and statistical analysis of key-performance-indicators (KPIs)

Fish larval samples were stained to reveal bone and cartilage^55^ and examined for the presence of skeletal abnormalities, following the terminology of Koumoundouros^12^ for vertebral and fin abnormalities, and Fragkoulis et al.^23^ for jaw-abnormalities. Observations were performed on both body sides, under a stereomicroscope (Olympus SZX 16). Abnormalities were recorded independently of their potential effect on fish external phenotype or recovery potential in the next developmental stages^28^. Light malformations of single skeletal elements (e.g., light shape alteration of epurals, pterygiophores or branchiostegal rays) were not included in the analysis. In addition to registering abnormality frequency, other examined KPIs included the frequency of fish with a normally inflated swimbladder (at 16 dph), the specific growth rate (SGR) and the homogeneity of fish TL (coefficient of variation, CV) at the quality control stage, survival rate (Sur) and tank productivity (Prd). SGR was calculated using the formula SGR = (lnTL_2 _− lnTL_1_)/(t_2_), where TL_2_ is the mean TL and t_2_ the age (dph) of the samples that were collected for quality control monitoring, and TL_1_ is the TL at hatching (2.5 mm^56^). The productivity index was calculated as the mean number of fish that survived (27–70 dph) divided by the volume (L) of the larval-rearing tank (fish/L).

Classification tree analysis (CTa) is considered a valuable method for identifying critical parameters that explain the variability of independent variables. CTa is a supervised non-parametric method, without any assumption requirements on data distribution, able to deal with high-order interactions and non-linear relationships. To explain variation of a single independent (response) variable, trees repeatedly split the data into more homogenous groups, using combinations of explanatory variables (numeric and/or categorical^47^). In the present study, independent variables (KPIs) were transposed into categorical variables, by coding their lower half values as "0" and the remaining (higher) as “1”. Monitored rearing parameters were checked for data completeness (DC, percentage of non-missing data entries), and those with a DC < 80% were excluded from the analysis (Table S3). In the remaining 150 primary and secondary variables (Table S2), blank entries were replaced with the average value of each specific hatchery. CTa was performed separately for each hatchery by means of SPSS v26 software. For each hatchery and KPI, multiple successive CTa was performed on the same dataset, after removing the variable which in the previous CTa was identified as an important predictor in the resulted right-sized tree (pruning on the basis of misclassification error). Since the learning dataset was small and no test dataset was available, the V-fold cross-validation option was applied to select the right-sized pruned tree, i.e., the smallest-sized, least complex, tree whose cross-validation cost does not differ considerably from minimum cross-validation costs^57^. The significance of the differences in the predictor variables between the "0" and "1" groups was tested by Mann–Whitney U statistic.

### Hatchery genetic structure

Genetic analysis was performed on eighteen (18) independently sampled larval batches of gilthead seabream larvae of 40–56 dph from the hatcheries participating in the KPI study. On average four batches per hatchery were used for genetic analysis. From each examined batch, a random sample of 10 larvae was anaesthetized and dipped in ethanol. Upon their arrival in the lab, all samples were anonymized and coded. DNA extraction was carried out based on Aljanabi’s protocol^58^. Genetic variability was evaluated using microsatellite analysis with validated primer sets; SauK140INRA, SaI12, Saimbb26 and Fd-78-H^59,60^. The primers were selected based on the results of our previous studies, according to their allelic richness and levels of observed heterozygosity (Tables S4, S5). Sequencing reads were performed in an ABI 3730XL and the output collected for genotype using GeneScan®. Pairwise Fst and Nei’s distances of the hatchery pairs, and the observed (HO), and the expected (HE) heterozygosity for the total of samples and per hatchery were calculated using the MSA 4.05 program^61^. Hatchery structure analysis was performed using Structure 2.3.4^62^ assuming K = 1 to K = 5 for individual hatcheries and K = 1 to K = 7 for the total of samples. For each K calculation, 5.000 repetitions were applied as burn-in followed by 50.000 repetitions after burn-in and 10 simulations. The best K value was determined using structure harvester^63^ based on Evanno’s method^64^.

### Ethical statement

During this study no experimentation with alive animals was performed. The examined biological material consisted exclusively of fixed samples taken during routine rearing procedures, in commercial finfish hatcheries, registered for aquaculture production in EU countries. Animal sampling followed routine procedures and samples were collected by a qualified staff member from standard production cycles. The legislation and measures implemented by the commercial producers complied with existing national and EU (Directive 63/2010) legislation (protection of animals kept for farming). Production and sampling, by an experienced staff member, were optimized to avoid unnecessary pain, suffering or injury.

## Supplementary Information

Supplementary Information.

## Data Availability

The data that support the findings of this study are available from the corresponding author upon reasonable request.

## References

[1] Sawada Y (2020). Positive phototaxis as the cause of jaw malformations in larval greater amberjack, *Seriola dumerili* (Risso, 1810): Mitigation by rearing in tanks with low-brightness walls. Aquac. Res..

[2] Sun J (2020). Skeletal anomalies in cultured golden pompano *Trachinotus ovatus* at early stages of development. Dis. Aquat. Organ..

[3] de Azevedo AM (2020). Skeletal anomalies in senegalese sole (*Solea senegalensis*, Kaup) fed with different commercial enriched artemia: A study in postlarvae and juveniles. Animals.

[4] Fernández I (2021). Skeletal development and deformities in tench (*Tinca tinca*): From basic knowledge to regular monitoring procedure. Animals.

[5] Fjelldal PG (2021). Skeletal deformities in wild and farmed cleaner fish species used in Atlantic salmon *Salmo salar* aquaculture. J. Fish Biol..

[6] Kousoulaki K (2021). Technical feed quality influences health, digestion patterns, body mineralization and bone development in farming of the stomachless cleaner fish ballan wrasse (*Labrus bergylta*). Anim. Feed Sci. Technol..

[7] Fragkoulis S (2017). Saddleback syndrome in European sea bass *Dicentrarchus labrax* (Linnaeus, 1758): Anatomy, ontogeny and correlation with lateral-line, anal and pelvic fin abnormalities. J. Fish Dis..

[8] Riera-Heredia N, Vélez EJ, Gutiérrez J, Navarro I, Capilla E (2019). Gene expression analyses in malformed skeletal structures of gilthead sea bream (*Sparus aurata*). J. Fish Dis..

[9] Fragkoulis S, Economou I, Moukas G, Koumoundouros G, Batargias C (2020). Caudal fin abnormalities in Gilthead seabream (*Sparus aurata* L.) have a strong genetic variance component. J. Fish Dis..

[10] Holm H (2020). A pathomorphological description of cross-stitch vertebrae in farmed Atlantic salmon (*Salmo salar* L.). Aquaculture.

[11] Fraser TWK, Hansen TJ, Sambraus F, Fjelldal PG (2021). Vertebral deformities in interspecific diploid and triploid salmonid hybrids. J. Fish Biol..

[12] Koumoundouros G (2010). Morpho-anatomical abnormalities in Mediterranean marine aquaculture. Rec. Adv. Aquac. Res..

[13] Izquierdo MS (2013). Effects of dietary DHA and α-tocopherol on bone development, early mineralisation and oxidative stress in *Sparus aurata* (Linnaeus, 1758) larvae. Br. J. Nutr..

[14] Fernández I (2008). Larval performance and skeletal deformities in farmed gilthead sea bream (*Sparus aurata*) fed with graded levels of Vitamin A enriched rotifers (*Brachionus plicatilis*). Aquaculture.

[15] Georga I (2011). Effect of vitamin A on the skeletal morphogenesis of European sea bass, *Dicentrarchus labrax* (Linnaeus, 1758). Aquac. Res..

[16] Printzi A (2021). Balancing between Artemia and microdiet usage for normal skeletal development in zebrafish (*Danio rerio*). J. Fish Dis..

[17] Kourkouta C (2021). Long-lasting effects of early temperature exposure on the swimming performance and skeleton development of metamorphosing Gilthead seabream (*Sparus aurata* L.) larvae. Sci. Rep..

[18] Cobcroft JM, Battaglene SC (2009). Jaw malformation in striped trumpeter *Latris lineata* larvae linked to walling behaviour and tank colour. Aquaculture.

[19] Sawada Y, Honryo T, Agawa Y, Kurata M (2018). Teratogenic effects of isolated and combined short-term hypercapnia and hypoxia on red sea bream (*Pagrus major*) embryos. Aquac. Res..

[20] Koumoundouros G, Divanach P, Kentouri M (2001). The effect of rearing conditions on development of saddleback syndrome and caudal fin deformities in *Dentex dentex* (L.). Aquaculture.

[21] Izquierdo MS, Socorro J, Roo J (2010). Studies on the appearance of skeletal anomalies in red porgy: Effect of culture intensiveness, feeding habits and nutritional quality of live preys. J. Appl. Ichthyol..

[22] Negrín-Báez D (2015). Inheritance of skeletal deformities in gilthead seabream (*Sparus aurata*) – lack of operculum, lordosis, vertebral fusion and LSK complex1. J. Anim. Sci..

[23] Fragkoulis S, Batargias C, Kolios P, Koumoundouros G (2018). Genetic parameters of the upper-jaw abnormalities in Gilthead seabream *Sparus aurata*. Aquaculture.

[24] Boglione C (2013). Skeletal anomalies in reared European fish larvae and juveniles. Part 1: normal and anomalous skeletogenic processes. Rev. Aquac..

[25] Fragkoulis S, Printzi A, Geladakis G, Katribouzas N, Koumoundouros G (2019). Recovery of haemal lordosis in Gilthead seabream (*Sparus aurata* L.). Sci. Rep..

[26] Beraldo P, Canavese B (2011). Recovery of opercular anomalies in gilthead sea bream, *Sparus aurata* L. morphological and morphometric analysis. J. Fish Dis..

[27] Cavrois-Rogacki T, Drabikova L, Migaud H, Davie A (2021). Deformities prevalence in farmed ballan wrasse (*Labrus bergylta*) in relation to hatchery origin and life stage. Aquaculture.

[28] Fragkoulis S, Koumoundouros G (2021). Simple morphometrics for predicting lordosis-induced deviations of body shape in reared Gilthead seabream (*Sparus aurata* L.). J. Fish Dis..

[29] Loizides M, Georgiou AN, Somarakis S, Witten PE, Koumoundouros G (2014). A new type of lordosis and vertebral body compression in Gilthead seabream, *Sparus aurata* L.: aetiology, anatomy and consequences for survival. J. Fish Dis..

[30] Cobcroft JM, Battaglene SC (2013). Skeletal malformations in Australian marine finfish hatcheries. Aquaculture.

[31] Prestinicola L (2013). Environmental conditioning of skeletal anomalies typology and frequency in gilthead seabream (*Sparus aurata* L., 1758) Juveniles. PLoS ONE.

[32] Karaiskou N, Triantafyllidis A, Katsares V, Abatzopoulos TJ, Triantaphyllidis C (2009). Microsatellite variability of wild and farmed populations of *Sparus aurata*. J. Fish Biol..

[33] Loukovitis D (2012). Genetic variation in farmed populations of the gilthead sea bream *Sparus aurata* in Greece using microsatellite DNA markers. Aquac. Res..

[34] Polovina E-S, Kourkouni E, Tsigenopoulos CS, Sanchez-Jerez P, Ladoukakis ED (2020). Genetic structuring in farmed and wild Gilthead seabream and European seabass in the Mediterranean Sea: Implementations for detection of escapees. Aquat. Living Resour..

[35] Kihara M, Ogata S, Kawano N, Kubota I, Yamaguchi R (2002). Lordosis induction in juvenile red sea bream, *Pagrus major*, by high swimming activity. Aquaculture.

[36] Sfakianakis DG (2006). Environmental determinants of haemal lordosis in European sea bass, *Dicentrarchus labrax* (Linnaeus, 1758). Aquaculture.

[37] Printzi A (2021). Exercise-induced lordosis in zebrafish *Danio rerio* (Hamilton, 1822). J. Fish Biol..

[38] Georgakopoulou E, Katharios P, Divanach P, Koumoundouros G (2010). Effect of temperature on the development of skeletal deformities in Gilthead seabream (*Sparus aurata* Linnaeus, 1758). Aquaculture.

[39] Palstra AP (2020). Physiological effects of water flow induced swimming exercise in seabream *Sparus aurata*. Front. Physiol..

[40] Villeneuve L, Gisbert E, Zambonino-Infante JL, Quazuguel P, Cahu CL (2005). Effect of nature of dietary lipids on European sea bass morphogenesis: implication of retinoid receptors. Br. J. Nutr..

[41] Gapasin RSJ, Bombeo R, Lavens P, Sorgeloos P, Nelis H (1998). Enrichment of live food with essential fatty acids and vitamin C: Effects on milkfish (*Chanos chanos*) larval performance. Aquaculture.

[42] Gapasin RSJ, Duray MN (2001). Effects of DHA-enriched live food on growth, survival and incidence of opercular deformities in milkfish (*Chanos chanos*). Aquaculture.

[43] Villeneuve L, Gisbert E, le Delliou H, Cahu CL, Zambonino-Infante JL (2005). Dietary levels of all-trans retinol affect retinoid nuclear receptor expression and skeletal development in European sea bass larvae. Br. J. Nutr..

[44] Villeneuve LAN, Gisbert E, Moriceau J, Cahu CL, Infante JLZ (2006). Intake of high levels of vitamin A and polyunsaturated fatty acids during different developmental periods modifies the expression of morphogenesis genes in European sea bass (*Dicentrarchus labrax*). Br. J. Nutr..

[45] Sfakianakis DG, Koumoundouros G, Divanach P, Kentouri M (2004). Osteological development of the vertebral column and of the fins in *Pagellus erythrinus* (L. 1758). Temperature effect on the developmental plasticity and morpho-anatomical abnormalities. Aquaculture.

[46] Mazurais D (2009). Optimal levels of dietary vitamin A for reduced deformity incidence during development of European sea bass larvae (*Dicentrarchus labrax*) depend on malformation type. Aquaculture.

[47] De’ath G, Fabricius KE (2000). Classification and regression trees: A powerful yet simple technique for ecological data analysis. Ecology.

[48] Gjerde B, Pante MJR, Baeverfjord G (2005). Genetic variation for a vertebral deformity in Atlantic salmon (*Salmo salar*). Aquaculture.

[49] Bardon A (2009). What is the heritable component of spinal deformities in the European sea bass (*Dicentrarchus labrax*)?. Aquaculture.

[50] Nguyen NH, Whatmore P, Miller A, Knibb W (2016). Quantitative genetic properties of four measures of deformity in yellowtail kingfish *Seriola lalandi* Valenciennes, 1833. J. Fish Dis..

[51] Batargias, C. Genetics of seabream (*Sparus aurata*). Study of microsatellites and their use for the estimation of genetic parameters of growth and other quantitative characters. Ph.D. dissertation, University of Crete, Heraklion, Crete, Greece. (In Greek with English summary) (1998).

[52] García-Celdrán M (2016). Estimates of heritabilities and genetic correlations of skeletal deformities and uninflated swimbladder in a reared gilthead sea bream (*Sparus aurata* L.) juvenile population sourced from three broodstocks along the Spanish coasts. Aquaculture.

[53] Bertolini F (2021). A comparative whole genome sequencing analysis identified a candidate locus for lack of operculum in cultivated gilthead seabream (*Sparus aurata*). Anim. Genet..

[54] Maroso F (2021). Genome-wide analysis clarifies the population genetic structure of wild gilthead sea bream (*Sparus aurata*). PLoS ONE.

[55] Walker M, Kimmel C (2007). A two-color acid-free cartilage and bone stain for zebrafish larvae. Biotech. Histochem..

[56] Polo A, Yúfera M, Pascual E (1991). Effects of temperature on egg and larval development of *Sparus aurata* L. Aquaculture.

[57] Breiman L, Friedman JH, Olshen RA, Stone CJ (1984). Classification and Regression Trees.

[58] Aljanabi S, Martinez I (1997). Universal and rapid salt-extraction of high-quality genomic DNA for PCR- based techniques. Nucl. Acids Res..

[59] Navarro A (2008). Development of two new microsatellite multiplex PCRs for three sparid species: Gilthead seabream (*Sparus auratus* L.), red porgy (*Pagrus pagrus* L.) and redbanded seabream (P. *auriga*, Valenciennes, 1843) and their application to paternity studies. Aquaculture.

[60] Negrín-Báez D (2015). A set of 13 multiplex PCRs of specific microsatellite markers as a tool for QTL detection in gilthead seabream (*Sparus aurata* L.). Aquac. Res..

[61] Dieringer D, Schlötterer C (2003). Microsatellite analyser (MSA): A platform independent analysis tool for large microsatellite data sets. Mol. Ecol. Notes.

[62] Pritchard JK, Stephens M, Donnelly P (2000). Inference of population structure using multilocus genotype data. Genetics.

[63] Earl DA, von Holdt BM (2012). STRUCTURE HARVESTER: A website and program for visualizing STRUCTURE output and implementing the Evanno method. Conserv. Genet. Resour..

[64] Evanno G, Regnaut S, Goudet J (2005). Detecting the number of clusters of individuals using the software STRUCTURE: A simulation study. Mol. Ecol..

