# Supplementary material for: Variability of key-performance-indicators in commercial gilthead seabream hatcheries
Source: Sci Rep. 2022 Oct 25;12:17896. doi: 10.1038/s41598-022-23008-z (PMC9596474; doi:10.1038/s41598-022-23008-z)
Supplement: Supplementary file 1 — Supplementary Information. [file 41598_2022_23008_MOESM1_ESM.docx]

**Variability of Key-Performance-Indicators in commercial gilthead seabream hatcheries**

Chara Kourkouta^a^, Andreas Tsipourlianos^b^, Deborah M. Power^c^, Katerina A. Moutou^b^,
George Koumoundouros^a,*^

^a^ Biology Department, University of Crete, Vasilika Vouton, 70013, Heraklion, Crete, Greece

^b^ Department of Biochemistry and Biotechnology, University of Thessaly, 41500, Larissa, Greece

^c^ Centre of Marine Sciences (CCMAR), University of Algarve, Faro, Portugal

*, corresponding author, E-mail address: [gkoumound@uoc.gr](mailto:gkoumound@uoc.gr)

**Table S1**. Rearing parameters which were included in the prepared list for data recording.

| Rearing phase | Parameters |
| --- | --- |
| Broodstock management | Biotic: mean age and size of breeders, sex ratio, fertilization rate, egg stage at collection, stocking density.  Abiotic: water temperature, oxygen concentration, salinity, water renewal rate and ph at different steps of this rearing phase. Also, tank characteristics, collector characteristics, products for egg disinfection.  Nutritional: feeding rate, feed type. |
| Embryonic & yolk-sac stage | Biotic: stocking density  Abiotic: water temperature, oxygen concentration, salinity, water renewal rate, pH, light intensity and photoperiod at different steps of this rearing phase. Also, tank characteristics. |
| Larval rearing, weaning and pre-growing | Biotic: initial stocking density, stage at stocking, survival (at 35 dph and at fish transfer to the next phase), observations on fish behavior, details on the use of background phytoplankton (e.g. species, concentration, origin, duration), fish size and swimbladder inflation rate at different steps of this rearing phase.  Abiotic: water temperature, oxygen concentration, salinity, water renewal rate, pH, ammonia, water aeration methodology, light intensity and photoperiod at different steps of this rearing phase. Also, tank characteristics, tank disinfection methodology, microbial-control methodology, tank cleaning methodology.  Nutritional: feeding rates and feed types (at different steps of this rearing phase), products and methodology used for enrichment of live feed, methodology of live-feed transition. |

**Table S2**. Rearing parameters which were included in the prepared list for data recording.

| **Var s/n** | **Variables** |
| --- | --- |
| 1 | Use of eggs of different age |
| 2,3 | Mean age (year) and weight (Kg) of breeders |
| 4 | Broodstock Sex Ratio (F/M) |
| 5-10 | Broodstock feed, feeding frequency & rate (before & during spawning) |
| 11 | Time at egg collection |
| 12-13 | Water temperature & salinity (egg collector) |
| 14 | Volume of the egg collector (L) |
| 15 | Egg-disinfection product |
| 16 | Use of egg incubator |
| 17-19 | Volume (m^3^), shape and color of the larval-rearing tank |
| 20 | Number of larval-rearing tanks in the same area |
| 21 | Number of air diffusers in the larval-rearing tank |
| 22 | Duration of "Dry Period" (d) |
| 23-26 | Tank-disinfection agent & chemical therapies (duration, agent, frequency) during the larval rearing |
| 27 | Frequency of tank siphon-cleaning |
| 28 | Ontogenetic stage at stocking (eggs, y-s larvae) |
| 29 | Initial Stocking Density (ind/L) |
| 30-38 | Age (dph) at first & last plankton provision (algae, rotifers, Artemia instar I & II), and at weaning onset |
| 39-46 | Quantity of rotifers provided (3, 4, 5, 3-7, 8-12, 13-17, 18-22, 23-27 dph) |
| 47-51 | Quantity of Artemia instar-I nauplii provided (6-10, 11-15, 16-20, 21-25, 26-30 dph) |
| 52-57 | Quantity of Artemia instar-II nauplii provided (15, 20, 25, 30, 15-25, 26-36 dph) |
| 58-63 | Quantity of dry-feed provided (6-10, 11-15, 16-20, 21-25, 26-30, 31-35 dph) |
| 64-66 | Total quantity of rotifers & Artemia (instar I & II) provided up to 35 dph |
| 67-68 | Micro-algae used (species, single use or combination) |
| 69-75 | Product names of Artemia, starter diets, enrichment-media and Artemia-bacteriostatics |
| 76-79 | Daily number of plankton provisions (algae, rotifers, Artemia instar-I and -II) |
| 80-82 | Live-feed (rotifers, Artemia instar-I and -II) adjustment levels (ind/mL) |
| 83-91 | Mean water temperature (spawning, 1 dpf, 0-4, 5-9, 10-14, 15-19, 20-24, 25-29, 35 dph) |
| 92-97 | SD of water temperature (0-4, 5-9, 10-14, 15-19, 20-24, 25-29 dph) |
| 98-102 | Daily water-temperature difference (1, 2, 3, 4, 5 dph) |
| 103-104 | Cumulative daily water-temperature difference (0-10 & 11-21 dph) |
| 105-110 | Mean oxygen levels (0-4, 5-9, 10-14, 15-19, 20-24, 25-29 dph) |
| 111-116 | SD of oxygen levels (0-4, 5-9, 10-14, 15-19, 20-24, 25-29 dph) |
| 117-119 | Cumulative daily difference of oxygen-levels (3-10, 11-20 & 21-30 dph) |
| 120-124 | pH (4, 8, 12, 16, 20 dph) |
| 125-130 | Water exchange rate (0, 4, 12, 20, 30, 35 dph) (% of tank volume per hour) |
| 131-137 | Water source and treatment (UV, ozon, RAS, degassing, mechanical filtration) |
| 138-143 | Photoperiod (0, 8, 16, 20, 25, 30 dph) (hr/d) |
| 144-145 | Light intensity at the center & periphery of the larval-rearing tank (8 dph) |
| 146-150 | Salinity (0, 4, 12, 20, 30 dph) |

**Table S3**. Rearing parameters which were excluded from the analysis because of low data completeness (DC<80%, percentage of non-missing data entries).

| **Variables** |
| --- |
| Oxygen concentration and pH in the egg-collector |
| Volume of the egg incubator |
| Oxygen concentration, temperature. pH, salinity and light intensity in the egg-incubator |
| Depth and diameter of the larval-rearing tank |
| Survival rate at 35 dph |
| Quantity of rotifers provided (28, 29, 30 dph) |
| Algae adjustment levels (10^3^ cells/ml) |
| Water temperature (40, 50 dph) |
| pH (0, 35, 40, 60 dph) |
| Light intensity at the center & periphery of the larval-rearing tank (0, 4, 12, 16, 20, 25, 30 dph) |
| Salinity (4, 12, 20, 30, 35, 40, 60 dph) |
| Fish length (12, 25, 30, 50 dph) |

**Choice of Microsatellite Markers**

The genetic analysis in the present study was performed using microsatellite primer sets validated in previous studies (Navarro et al., 2008; Negrın-Baez et al., 2014). The choice of microsatellite markers used was based on knowledge gained through commercial projects, the results of which remain unpublished in agreement with non-disclosure agreements governing the project implementation. In those projects, the genetic variation of 731 commercially farmed fish from eight (8) populations was estimated based on a set of eight (8) validated microsatellite markers. For those markers we identified the number of alleles and the percentage of heterozygosity (Tables S4 & S5). However, a considerable number of rare alleles was identified for markers SAI14 and SAI21. Based on this knowledge, the four markers (SAIMBB26, FD78H, SAI12, SAUK140INRA) selected for use in the present study fulfilled the following combination of criteria: a) a high number of alleles, *and* b) no presence of rare alleles, *and* c) high percentage of heterozygosity.

**Table S4. Number of alleles identified per population and per marker***

| No of Population | No of individuals | SAGT41B | **SAIMBB26** | SAU97INRA | **FD78H** | **SAI12** | SAI14 | SAI21 | **SAUK140INRA** |
| --- | --- | --- | --- | --- | --- | --- | --- | --- | --- |
| 1 | 65 | 15 | 15 | 12 | 15 | 14 | 17 | 16 | 16 |
| 2 | 67 | 15 | 19 | 8 | 15 | 9 | 24 | 21 | 9 |
| 3 | 80 | 20 | 21 | 11 | 12 | 15 | 17 | 25 | 12 |
| 4 | 79 | 16 | 16 | 12 | 15 | 13 | 19 | 18 | 9 |
| 5 | 102 | 16 | 16 | 11 | 13 | 17 | 15 | 18 | 8 |
| 6 | 142 | 18 | 19 | 12 | 16 | 17 | 21 | 17 | 12 |
| 7 | 91 | 19 | 22 | 11 | 15 | 15 | 16 | 21 | 13 |
| 8 | 105 | 14 | 18 | 13 | 14 | 23 | 19 | 16 | 12 |

* Brown, R.C. *et al.* Additional microsatellites for *Sparus aurata* and cross-species amplification within the Sparidae family. *Mol.Ecol.Res.* **5,** 605-607 (2005); Castro, J. *et al.* A microsatellite marker tool for parentage assessment in gilthead seabream (*Sparus aurata*). *Aquaculture* **272**, 210-216 (2007); Navarro, A. *et al.* Development of two new microsatellite multiplex PCRs for three sparid species: Gilthead seabream (*Sparus auratus* L.), red porgy (*Pagrus pagrus* L.) and redbanded seabream (*P. auriga*, Valenciennes, 1843) and their application to paternity studies. *Aquaculture* **285**, 30-37 (2008); Karaiskou, N. *et al*. Microsatellite variability of wild and farmed populations of Sparus aurata. *J Fish Biol.* **74**, 816-1825 (2009); Negrín-Báez, D. *et al.* A set of 13 multiplex PCRs of specific microsatellite markers as a tool for QTL detection in gilthead seabream (*Sparus aurata* L.). *Aquac. Res.* **46**, 45-58 (2015).

**Table S5. Heterozygosity rates per population and per marker; mean heterozygosity rates between populations**

| No of Population | SAGT41B | **SAIMBB26** | SAU97INRA | **FD78H** | **SAI12** | SAI14 | SAI21 | **SAUK140INRA** |
| --- | --- | --- | --- | --- | --- | --- | --- | --- |
| 1 | 97 | 89 | 73 | 77 | 83 | 85 | 26 | 78 |
| 2 | 97 | 97 | 98 | 100 | 100 | 98 | 88 | 100 |
| 3 | 73 | 66 | 48 | 96 | 85 | 66 | 55 | 85 |
| 4 | 95 | 87 | 78 | 86 | 86 | 97 | 41 | 77 |
| 5 | 98 | 97 | 95 | 99 | 99 | 98 | 53 | 98 |
| 6 | 72 | 88 | 90 | 99 | 80 | 86 | 47 | 94 |
| 7 | 45 | 78 | 95 | 96 | 85 | 89 | 51 | 98 |
| 8 | 64 | 92 | 88 | 90 | 76 | 76 | 17 | 94 |
| **mean He** | 80 | **86** | 83 | **92** | **86** | 86 | 47 | **90** |


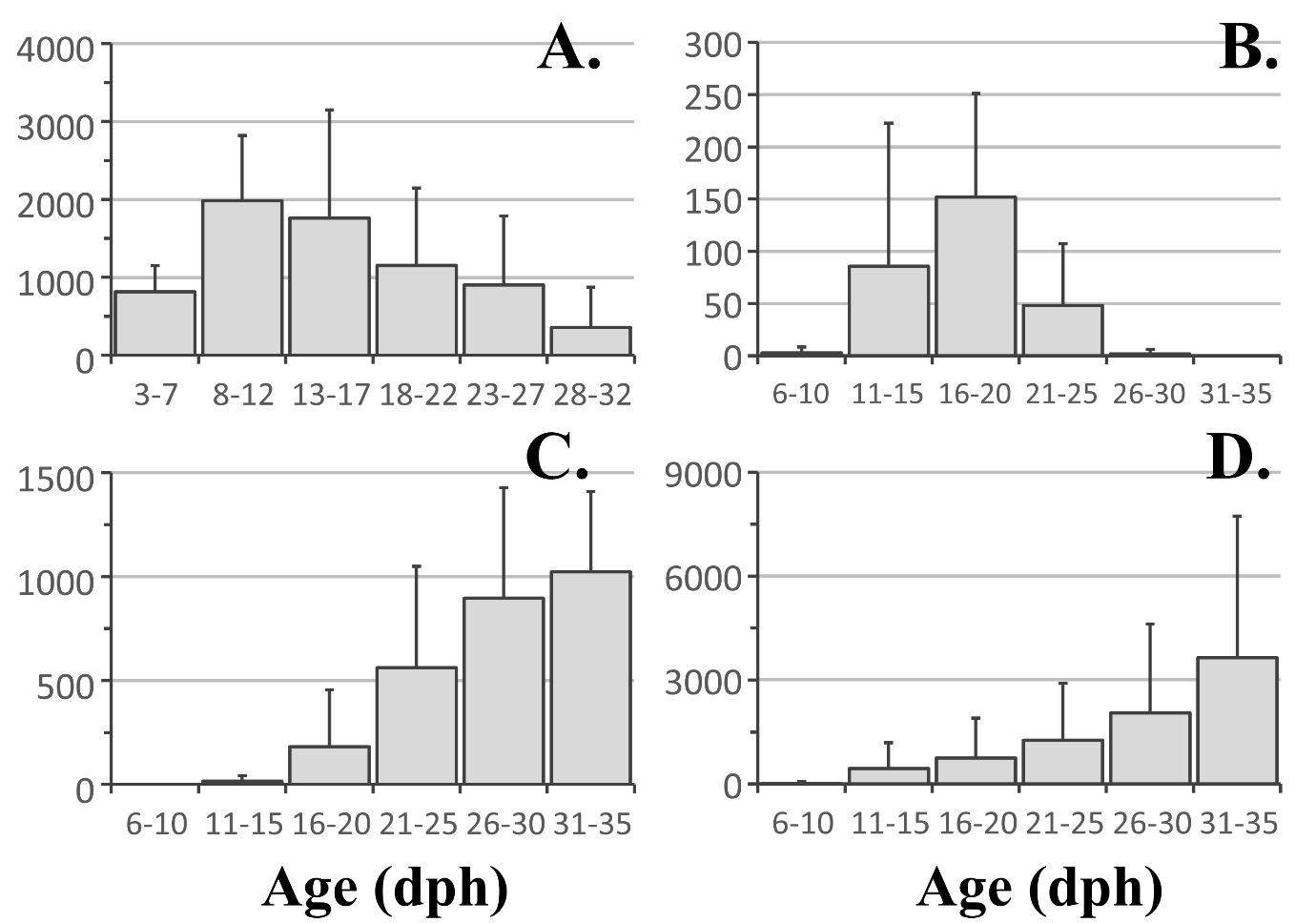


**Fig S1**. Mean quantities of live and dry feeds provided to each larval population during the first 35 post-hatching days (dph). **A.** Rotifers (x10^6^). **B.** Artemia instar I nauplii (x10^6^). **C.** Artemia instar II nauplii (x10^6^). **D.** Commercial dry feed (g). Error bars equal to 1 SD.


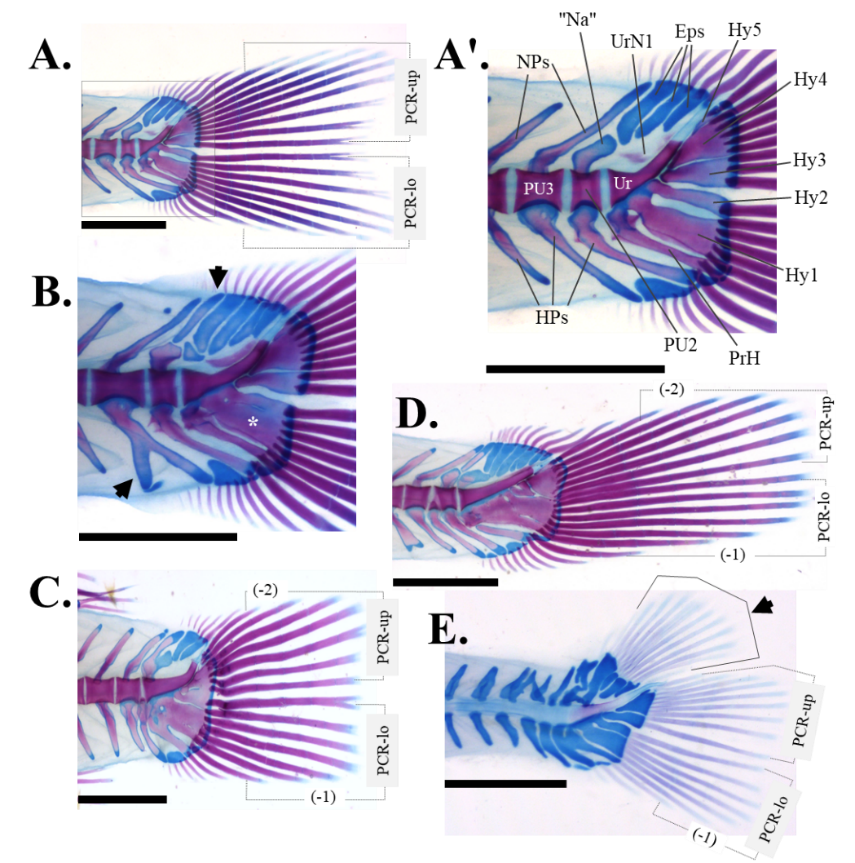


**Fig. S2**. Variability of caudal-fin abnormalities in the examined samples. **A**. Normal. **A'**. Inset of figure A, showing in detail the internal anatomy of the caudal-fin. **B**. Light abnormalities (arrows) of the caudal fin. Asterisk indicates the abnormal fusion of hypurals 1 and 2. **C-E**. Severe abnormalities of the caudal-fin (PCRs shortening, C; fin stricture, D; fin duplication, E), associated with multiple abnormalities of the fin supporting elements. Eps, epurals. HPs, haemal processes. Arrow in E indicated the extra-numerous PCRs. Hy1-Hy5, hypurals 1-5. "Na", modified neural arch. NPs, neural processes. PCR-lo, lower principal caudal-fin rays. PCR-up, upper principal caudal-fin rays. PrH, parhypural. PU2 and PU3, pre-ural centrum 2 and 3 respectively. Ur, urostyle. UrN1, uroneural 1. Numbers in brackets indicate the difference of rays number from the normal (9 PCR-up and 8 PCR-lo). Scale bars equal to 1 mm.


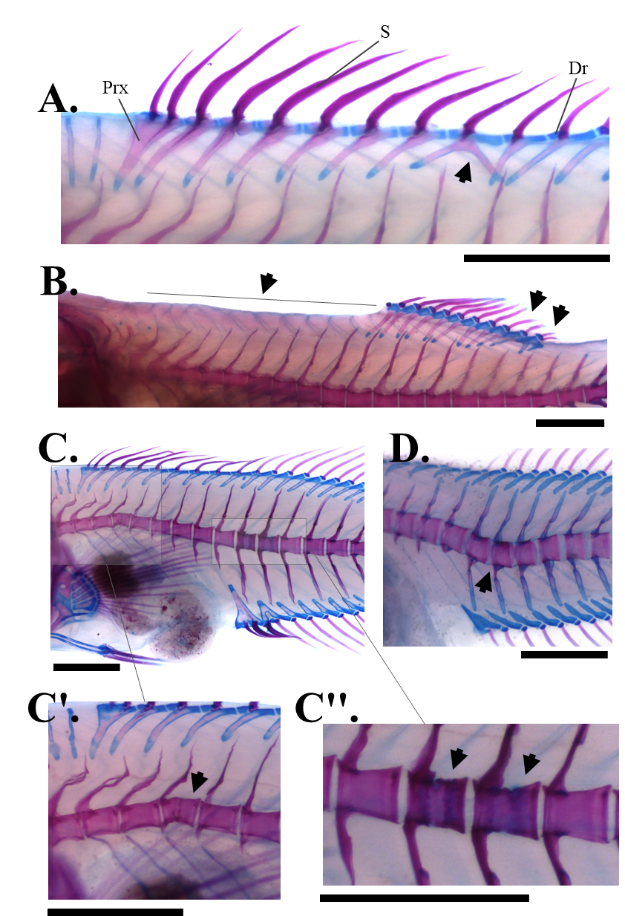


**Fig. S3**. Variability of dorsal-fin and vertebral abnormalities in the examined samples. **A**. Light abnormality (arrow) of the dorsal fin. **B**. Saddleback syndrome. Arrows indicate the missing pterygiophores and spines (anterior) or abnormal rays (posterior). **C-C"**. Light kyphosis (C') and abnormalities of centra without associated axis deviations (C"). **D**. Haemal lordosis (arrow). Scale bars equal to 1 mm.
